# Formative Work to Develop a Tailored HIV Testing Smartphone App for Diverse, At-Risk, HIV-Negative Men Who Have Sex With Men: A Focus Group Study

**DOI:** 10.2196/mhealth.6178

**Published:** 2016-11-16

**Authors:** Jason W Mitchell, Maria Beatriz Torres, Jennifer Joe, Thu Danh, Bobbi Gass, Keith J Horvath

**Affiliations:** ^1^ Office of Public Health Studies University of Hawai’i at Mānoa Honolulu, HI United States; ^2^ Communication Studies Gustavus Adolphus College Saint Peter, MN United States; ^3^ School of Public Health University of Minnesota Minneapolis, MN United States

**Keywords:** smartphone apps, mHealth, HIV testing, HIV-negative men who have sex with men, men who have sex with men, MSM

## Abstract

**Background:**

Although gay, bisexual, and other men who have sex with men (MSM) are disproportionately affected by human immunodeficiency virus (HIV) infection, few test for HIV at regular intervals. Smartphone apps may be an ideal tool to increase regular testing among MSM. However, the success of apps to encourage regular testing among MSM will depend on how frequently the apps are downloaded, whether they continue to be used over months or years, and the degree to which such apps are tailored to the needs of this population.

**Objective:**

The primary objectives of this study were to answer the following questions. (1) What features and functions of smartphone apps do MSM believe are associated with downloading apps to their mobile phones? (2) What features and functions of smartphone apps are most likely to influence MSM’s sustained use of apps over time? (3) What features and functions do MSM prefer in an HIV testing smartphone app?

**Methods:**

We conducted focus groups (n=7, with a total of 34 participants) with a racially and ethnically diverse group of sexually active HIV-negative MSM (mean age 32 years; 11/34 men, 33%, tested for HIV ≥10 months ago) in the United States in Miami, Florida and Minneapolis, Minnesota. Focus groups were digitally recorded, transcribed verbatim, and deidentified for analysis. We used a constant comparison method (ie, grounded theory coding) to examine and reexamine the themes that emerged from the focus groups.

**Results:**

Men reported cost, security, and efficiency as their primary reasons influencing whether they download an app. Usefulness and perceived necessity, as well as peer and posted reviews, affected whether they downloaded and used the app over time. Factors that influenced whether they keep and continue to use an app over time included reliability, ease of use, and frequency of updates. Poor performance and functionality and lack of use were the primary reasons why men would delete an app from their phone. Participants also shared their preferences for an app to encourage regular HIV testing by providing feedback on test reminders, tailored testing interval recommendations, HIV test locator, and monitoring of personal sexual behaviors.

**Conclusions:**

Mobile apps for HIV prevention have proliferated, despite relatively little formative research to understand best practices for their development and implementation. The findings of this study suggest key design characteristics that should be used to guide development of an HIV testing app to promote regular HIV testing for MSM. The features and functions identified in this and prior research, as well as existing theories of behavior change, should be used to guide mobile app development in this critical area.

## Introduction

Approximately 1.2 million people are living with human immunodeficiency virus (HIV) in the United States, and 1 in 8 individuals is unaware of their infection [1]. Despite advances in antiretroviral therapy and steady prevention efforts, gay, bisexual, and other men who have sex with men (MSM) continue to be disproportionately affected by HIV. In 2014, MSM accounted for 82% of diagnoses of HIV infections among males, despite representing only 2% of the US population [2,3].

Given the HIV burden among MSM, the Centers for Disease Control and Prevention recommends that all sexually active MSM test for HIV at least annually, with more frequent testing for those who engage in high-risk behaviors (eg, condomless anal sex or drug use during sex) [4]. Studies show that nearly all MSM in the United States have tested for HIV in their lifetime, and approximately two-thirds have done so in the past year [5]. A study of young MSM residing in 5 US cities (Baltimore, Los Angeles, Miami, New York City, and San Francisco) found that 62% of participants had been tested in 2011, with increases in rates of HIV testing since the mid-1990s [6]. However, consistent and repeated testing for HIV (ie, testing for HIV at regular intervals) is needed to reduce the onward transmission of HIV associated with not knowing one’s status and, if HIV-positive, to reap the benefits of prompt antiretroviral therapy. Repeated HIV testing appears to be less common among MSM. A study showed that only half of sexually active HIV-negative MSM in concordant primary relationships tested for HIV at least annually (21% tested 2 or more times a year and 29% tested annually), while the remainder of the sample tested less frequently [7]. In the same study, 20% of men never tested for HIV while in their current relationship. Interventions encouraging repeated HIV testing among MSM are needed to address this ongoing need.

Mobile technologies have expanded dramatically in recent years, mirroring increased rates of mobile phone ownership. Ownership of mobile phones with advanced capabilities (referred to here as smartphones), such as those that allow access to the Internet and use apps, grew from 35% in 2011 to 64% in 2015 [8]. Smartphone ownership is particularly high among young adults (18- to 29-year-olds; 85%), and is higher among black (70%) and Hispanic (71%) US adults than among their white peers (61%) [8]. MSM were early adopters of technology [9], including the use of smartphone apps to sexually and socially connect with other MSM [10]. Because mobile device ownership and use has steadily risen over the years 2011-2015 [11], the use of apps and other mobile-based interventions is promising.

Although HIV prevention and treatment technologies that leverage technology are widespread [12,13], further development and testing of smartphone-based app interventions targeting MSM is needed. This need is relevant because MSM—particularly black and Latino MSM—remain disproportionately affected by HIV compared with their white counterparts. However, a review of available HIV and AIDS smartphone apps on the Google Play and Apple stores as of May 2015 found that only 7% of 285 available apps specifically targeted MSM [14]. Schnall and colleagues [15] applied the Information Systems Research (ISR) framework [16] to develop a smartphone app for HIV prevention that meets the needs of high-risk MSM. The ISR framework consists of 3 interrelated cycles: (1) a relevance cycle, (2) a rigor cycle, and (3) a design cycle. First, Schnall and colleagues [15] conducted focus groups with high-risk MSM to identify which features and functions were relevant for HIV prevention, which included (self-) information management, staying healthy, HIV testing, a chat/communication function, and resources. Through the rigor cycle of ISR, Schnall and colleagues reviewed mobile app use for HIV prevention with MSM and highlighted that the development and evaluation of smartphone apps for this purpose have not been well documented. The design cycle of ISR included the development phase of an HIV prevention app by incorporating findings obtained from the relevance and rigor cycles and eliciting feedback about the app from members of the target population (ie, high-risk MSM).

Focus groups have been used in several recent studies to increase the relevance of mobile apps tailored to high-risk MSM. First, for instance, Goldenberg and colleagues [17] recruited MSM (n=38) residing in Atlanta, Seattle, and rural US regions to obtain data about their preferences for an HIV prevention app. Across groups, men reported that HIV prevention smartphone apps should (1) have an educational component to guide their decisions for which test is best for them and prevention options; (2) be interactive and engaging with personalized feedback about their own sexual behaviors; (3) provide a social networking component with other MSM; (4) use language that is simple and understandable to the community; and (5) address privacy concerns by ensuring that the app is from a credible source and by having secure messaging features [17]. Second, Aliabadi and colleagues [18] used the information, motivation, and behavioral skills model to guide focus group discussions with high-risk MSM to better understand their preferences for an HIV prevention app. Key informational (eg, HIV testing and support group information), motivational (eg, addressing sexual encounters in which men intend to use condoms, but do not), and behavioral skill (eg, negotiating safer sex, understanding signs of HIV infection) needs were identified as critical content for their HIV prevention app [18].

Similar to the studies [15,17,18] described above, in our study we conducted focus groups with at-risk MSM to inform the subsequent development of an HIV-related smartphone app. However, this study expanded on the findings of prior studies in several important ways. First, our focus groups explored MSM’s use of and attitudes toward smartphone apps that they currently had on their mobile phones to better understand what features and functions they perceived to be associated with regular use of apps over time. Understanding why men may continue to use (or not use) certain apps over time may provide critical insights into how to design a sustainable mobile HIV testing app for MSM. Second, we recruited men living in Miami, Florida and Minneapolis, Minnesota to assess whether the findings in the earlier studies [15,17,18] were applicable to MSM living in other regions of the United States.

The overarching goal of this study was to elicit feedback about smartphone app use from HIV-negative MSM to apply these lessons toward the development of an engaging and sustainable HIV testing smartphone app. We established 3 primary objectives of these focus groups to meet our goal. First, we sought to understand what features and functions of smartphone apps MSM believed were associated with downloading apps to their mobile phones. Next, we asked MSM to reflect on what features and functions of smartphone apps they believed influenced them to sustain their use of apps on their phone over time. Finally, similar to prior studies [15,17-19], we asked men to describe what features and functions they would prefer to have in an HIV testing smartphone app.

## Methods

### Participants

We conducted 5 focus groups, 3 in Miami, Florida and 2 in Minneapolis, Minnesota, in January and February 2015. We recruited participants for the focus groups through targeted advertisements placed on Facebook, as well as by flyers placed at local community-based and AIDS service organizations and by word of mouth. Inclusion criteria for the study were self-reported and were (1) being a man 18 years of age or older, (2) being HIV-negative or having an unknown serostatus, (3) having had anal sex with another man in the past year, (4) owning a mobile phone with smartphone features (ie, global positioning system [GPS] technology, short message service, Internet browser capabilities, apps), and (5) being an English speaker. A total of 34 participants, 17 from Miami and 17 from Minneapolis, participated in the study.

### Procedures

The University of Minnesota and University of Miami institutional review boards approved all study procedures. The focus group questionnaire was developed by the research team and included questions, in a semistructured format, that explored participants’ experiences with smartphones and apps. Specifically, we asked participants to describe which apps they currently had on their smartphone. Then, men were asked to reflect on what general features and functions of smartphone apps they believed to influence their decisions to download, initiate use, and continue to use the app over time. Follow-up prompts were used to gain more insight into men’s decisions into smartphone app use if a particular topic (eg, usefulness, enjoyment, ease of use, security, cost, and peer influence) did not spontaneously emerge. Finally, participants were also asked to provide input about what features and functions that they would like to have in a smartphone app to encourage testing for HIV and other sexually transmitted infections.

All recruitment materials included a link to the study website where interested persons were welcomed and asked to complete eligibility screener items, from which we obtained sociodemographic and most recent HIV testing behavior data. We asked those who met eligibility criteria to provide consent. Focus groups were conducted in confidential settings. To maintain confidentiality and promote truthful answers to the focus group questions, participants were encouraged to use and refer to each other by their first name only. Focus group discussions lasted from 90 to 105 minutes. All focus groups were digitally recorded, transcribed verbatim, and deidentified for analysis.

### Data Analysis

A team of 3 independent research associates coded the focus group data. Several meetings were conducted to (1) train associates in qualitative coding and (2) ensure that associates could accurately and consistently identify emerging codes and patterns in a sample transcript, while following the same iterative process. Training was led by 1 of the associates (MBT) with extensive experience in qualitative coding. Analysis of the collected data followed the process of “interrogating, sorting and synthetizing interviews” [20]. Coding helps identify and categorize the meanings expressed by interview participants.

The first part of coding started by naming words, sentences, and paragraphs (codes). Then, the researcher grouped codes with similar meanings into larger categories of meaning. Codes and categories were refined, added, or eliminated as the heuristic process continued [21]. A constant comparison method (ie, grounded theory coding) was employed [22], with the focus group interviewees’ statements being continually examined and reexamined in terms of the themes revealed, points of consistency and of difference, and answers to the research questions. This process allowed us “to make implicit views, actions and processes more visible” [20].

To start, team members read the focus group transcripts several times. Working separately, each associate established a first cycle of coding. They identified codes (or themes) emerging from the data. The codes were noted in an Excel (Microsoft Corporation) spreadsheet, along with the representative interview quotes that referred to that theme. Associates then proceeded to cluster these themes and codes into categories or patterns. Next, the team met to revise and refine their coding scheme to prepare for the second cycle of coding. The Excel files of the 3 coders were compiled and compared to determine areas of agreement and disagreements. Areas of disagreement ranged from using different labels to name a code or a pattern, to some codes or patterns not being identified by all 3 associates. We also examined quotes that were classified differently by coders. Disagreements were discussed and resolved to reach full consensus. In that process, associates collectively refined some codes and patterns that represented similar meanings, making sure that all possible meanings were accurately captured and recorded. Finally, a master file was created to reflect the agreements reached about the themes.

## Results

As Table 1 shows, participants were, on average, 32 years of age; the sample’s age ranged between 18 and 56 years. The study sample was racially and ethnically diverse: 7 of 17 men (41%) in Minneapolis and 4 of 17 men (23%) in Miami self-reported as nonwhite, and 7 of 17 men (41%) in Miami self-reported as Hispanic. With respect to participant’s most recent HIV test, 13 of 17 men (76%) in Minneapolis had been tested within 6 months prior to study enrollment compared with 7 of 17 men (41%) in Miami. Of the 32 men who responded to the question about what type of smartphone they owned (not shown), 16 (50%) owned an iPhone, 14 (44%) owned an Android-based phone, and 2 (6%) owned another type of phone (eg, Microsoft-based phone).

As Table 2 shows, we organized the themes that emerged from the focus groups into 5 main categories, aligning with the general structure of the interview: (1) reasons to download an app, (2) reasons associated with downloading and using the app over time, (3) reasons associated with keeping and using the app over time, (4) reasons associated with deleting an app, and (5) preferences for features and functionality in an HIV testing app. Some themes that emerged from the focus group data were unique to participants’ downloading an app, keeping and using an app over time, or deleting an app. In contrast, a few themes transcended and appeared to influence whether participants downloaded and used the app over time, which are presented simultaneously below. In addition, themes that emerged from the data did not differ by location; the information and opinions shared by the men were similar among those in Miami and those in Minneapolis.

**Table 1:** Focus group sociodemographic characteristics.

Characteristics		Total (N=34)	Miami (n=17)	Minneapolis (n=17)
Age in years (mean)		32	34	31
**Race, n (%)**				
	White	23 (68)	13 (76)	10 (59)
	Black/African American	5 (15)	3 (18)	2 (12)
	Asian	2 (6)	1 (6)	1 (6)
	Native Hawaiian/Pacific Islander	1 (3)	0	1 (6)
	American Indian	1 (3)	0	1 (6)
	Other	2 (6)	0	2 (12)
**Ethnicity, n (%)**				
	Hispanic	9 (27)	7 (41)	2 (12)
	Non-Hispanic	25 (73)	10 (59)	15 (88)
**Most recent HIV^a^** **test, n (%)**				
	1-3 months ago	12 (35)	4 (24)	8 (47)
	4-6 months ago	8 (24)	3 (18)	5 (30)
	7-9 months ago	3 (9)	2 (12)	1 (6)
	10-12 months ago	5 (15)	4 (24)	1 (6)
	>1 year ago	3 (9)	2 (12)	1 (6)
	≥5 years ago	3 (9)	2 (12)	1 (6)

^a^HIV: human immunodeficiency virus.

**Table 2:** Themes, definitions, and participant endorsements of reasons to download, continue to use, and delete apps (N=34).

Theme		Definition	Participant endorsement, n (%)
**Reasons to download an app**			
	Cost	How much participants would spend on an app and whether cost would deter them from downloading it.	29 (85%) 18 preferred free 11 would pay
	Security	How secure an app is in terms of it having access to or protecting information.	25 (74%) 13 concerned 12 not concerned
	Efficiency	Discussion of whether the app enabled them to save time and added convenience in their life.	18 (53%)
**Reasons associated with downloading and using the app over time**			
	Usefulness and perceived necessity	Perception of the app to provide a certain function that helps to fill a certain need.	24 (71%)
	Influence by peers and posted reviews	Downloading and sustained use of certain apps because of reviews, rating, and word of mouth from peers.	Influence by others: 24 (71%) 17 yes, influenced 3 not influenced 4 sometimes
			Influence by reviews: 21 (62%) 15 yes, influenced 3 not influenced 3 sometimes
**Reasons associated with keeping** *and* **using the app over time**			
	Reliability	Discussion of whether the app is working properly and reliably compared with other apps.	4 (12%)
	Ease of use	The need for an app to be simple and easy to use to be kept.	13 (38%)
	Updates	Frequency at which the app would be updated.	16 (47%)
**Reasons associated with deleting an app**				
	Poor performance and functionality	App that does not work or needs too frequent updating, or has too many crashes.	11 (32%)
	Boredom and lack of use	Apps not being relevant anymore.	8 (24%)
**Preferences for HIV^a^** **testing App features and functionality**				
	HIV test reminders	Discussion of opinions about receiving reminders to get tested and preferences about format, frequency, and customization of those reminders.	34 (100%) 8 format 17 frequency 9 customization
	Recommended HIV testing intervals with dates	Discussion of receiving personalized, recommended testing intervals with specific dates of when to be tested next.	17 (50%)
	Details about HIV testing locations and HIV test locator	Sharing of opinions about wanting to know nearby locations to test and information about the testing sites.	23 (68%)
	Monitoring personal behaviors	Sharing of opinions about monitoring their own sexual behaviors.	21 (62%) 16 in favor 1 optional 4 against

^a^HIV: human immunodeficiency virus.

### Reasons to Download an App

#### Cost

The majority of participants preferred apps that were free:

It has to be free. I’m sorry. I’m on a budget. I can’t afford all of these apps, especially the really good ones.

33 years old, African American, Miami

However, a few indicated they did not mind paying a small amount (US $1-2) for an app, particularly if they thought it would be useful:

I’ve noticed that a big thing for me is barrier to entry, being cost primarily. If it’s a paid app I’m a lot less likely to download it to see what it is. If it’s a paid app, I want to know that it’s something, A) I’m going to use and B) I’m going to enjoy. If there’s a free version, even though it has ads and all that stuff, I’ll try that first. If I like it then I’ll pay for it. Having a cost associated with it, if it’s something that I’m not sure about, I definitely kind of stray away from that.

24 years old, white, Minneapolis

#### Security

Participants’ attitudes varied about the importance of the app being secure in terms of it having access to or protecting their information. For some, security was important to them:

I would say security is a big thing for me. It’s got to be secure for the information that I’m allowing it to have or giving it, especially if it’s a payment option or purchasing something. Also with the information that it utilizes from either other apps or from information I input, I want that to be secure. I make sure that it’s got security features that it’s a trusted app so before I download an app, it will run through the security feature on my phone

34 years old, white, Miami

Other men expressed less concern, although their concerns were heightened by the type and amount of personal information stored in the app:

I generally don’t worry about security all that much unless I’m entering a decent amount of personal information. As far as people seeing what’s on my phone, I really don’t [care]...if they really want to know what’s there then I’ll show them. It’s not a big deal. But my banking information, stuff like that, social security, those I’m quite protective of.

43 years old, white, Minneapolis

In contrast, others had little to no concerns about whether the app was secure:

I, to be honest, it doesn’t. That’s not something I really worry about at all. I guess I just don’t think about it.

28 years old, biracial Hispanic, Minneapolis

#### Efficiency

Participants shared that they were more likely to download an app if it enabled them to save time and added convenience in their life. For instance:

If an app gives me the ability to do something…more quickly…then I’ll download that app.

27 years old, biracial, Minneapolis

If it’s going to add a lot of convenience to something [then I’m more likely to download it].

19 years old, white, Miami

### Reasons Associated With Both Downloading and Using an App Over Time

#### Usefulness and Perceived Necessity

Another key influential factor was whether men perceived the app to help fill a particular need in their life; that is, the men would often ask themselves if the app performed a particular function that would be useful for them:

For myself, I tend to look at utility apps, productivity apps, banking apps, chat apps, or text—things like that that are very utility based; absolutely. That’s the deciding factor. It always come down to, is it going to be useful for me or is this just going to be another app.

37 years old, white, Miami

Men also questioned the need for the app, what the app could do for them (ie, usefulness), and the importance of having it over a given time period:

It’s really based on, for me, the importance of the app and what it can do for me today, tomorrow, and next week.

37 years old, African American, Miami

Similarly, men perceiving a need for the app for a particular purpose also contributed to their rationale for continuing to use the app over time:

I’ll continue to use an app if it still meets the criteria that cause me to download it in the first place. If that app continues to be something that’s useful then I’ll keep it. There are apps on my phone that I might use twice a year, but if I know I’ll probably use this, then I’ll keep it.

27 years old, biracial, Minneapolis

#### Influence by Peers and Posted Reviews

Peer influence and reviews posted by others who used a particular app were influential to men in deciding whether to download an app (or not) onto their smartphone. For some participants, peer influence was a primary influencer:

I think number one reason I would download something is because I heard about it from someone, or people talking about it [app]. Just hearing about something a lot of. Even if I don’t really know what is it, download and just check it out and delete if it it’s nothing that I need.

28 years old, biracial Hispanic, Minneapolis

And although this individual’s peers influenced his decision to download an app, he also read the reviews posted by others about the app:

I usually read five reviews before I download it [app] and it’s deterred me from downloading a few apps before.

28 years old, biracial Hispanic, Minneapolis

Participants also expressed how peers both positively and negatively influenced whether they continued to use the app over time. For example:

My friends, I have a group of friends, we all have an iPhone and we’re all in a group chat. We send different stuff to each other if we like it, if we don’t. If somebody says something wrong with it, of course, end of the day, it’s your opinion but if two or three people say it’s a problem…then usually we end up removing it collectively because it’s not really of use.

37 years old, African American, Miami

In contrast, others voiced that peers had absolutely no influence on their decision to continue to use the app:

No, not at all. I’m my own person. If I like it and it’s worth it for me, then I’ll continue to [use it].

56 years old, white Hispanic, Miami

### Other Reasons Associated With Keeping and Using an App Over Time

Other reasons associated with men keeping and using their apps over time (ie, 3 months or longer) pertained to whether they perceived the app to be reliable or easy to use, and how often the app was updated.

#### Reliability

Men expressed their expectations of the app needing to reliably work when they use it:

I would say ease of access and reliability. Huge thing. If it’s crashing every two seconds then it starts becoming, is this something that I really, really need or send a crash report and just hold off or whatever.

34 years old, white, Miami

#### Ease of Use

Men also identified that intuitive features and functions were important for them to continue using an app over time:

I think simplicity is really the one thing that attracts me the most to an app. If it’s not too complicated and it serves my need, then I will definitely continue to use it. As soon as it starts to introduce a lot of features that I don’t really need…I will probably start to think about downloading some other apps that are simple to use and that can still serve my purpose.

23 years old, Asian, Miami]

#### Updates

Similar to the influence of peers about downloading and sustained use of an app, the frequency at which an app is updated was also voiced in both a positive and negative frame, which was dependent on personal preference. One participant noted that

I think daily would be annoying.

34 years old, white, Miami

This was in contrast to another participant, who stated that

It’s not going to bother me too much, if it’s a few times a week or something [like that].

28 years old, biracial Hispanic, Minneapolis

### Reasons Associated With Deleting an App

The primary reasons why participants deleted apps on their smartphone were largely contributed by their expectations not being met about the app.

#### Poor Performance and Functionality

Some of those reasons pertained to the app performing poorly, as noted by these 2 participants:

If it keeps on shutting down on me, I’m going to stop [and delete it].

26 years old, white Hispanic, Miami

If it’s [a] horrible user interface and it’s clunky and I can’t figure it out within the first two minutes of downloading the app, I’m just going to delete it.

24 years old, white, Minneapolis

#### Boredom and Lack of Use

Some men noted that being bored with an app and not using it for a while were reasons leading them to delete it:

If I’m not using it at all, I have a rule with myself. If I haven’t used it in two months I’ll delete it.

18 years old, white, Minneapolis

Boredom with the app was keenly expressed by this participant:

One day I’ll just wake up and say, I’m going to take a break from this one and I just [delete it]…when I get tired of it or bored.

56 years old, white Hispanic, Miami

### Preferences for HIV Testing App Features and Functionality

The latter part of the focus group discussion pertained to exploring participants’ attitudes about potential components for a future HIV testing app. The specific components explored were HIV test reminders, recommended testing intervals with dates, details about testing locations and a HIV test locator, and monitoring personal behaviors.

#### HIV Test Reminders

The discussion about HIV test reminders centered on 3 primary components: format, frequency, and customization. For format, a few participants preferred receiving the reminders via text, while others indicated they wanted the notifications sent through the app:

I would say just have it send it through the app itself. It will pop up with the little thing that says…

49 years old, white Hispanic, Miami

The frequency in which men wanted to receive the HIV test reminders varied and ranged from daily, weekly, and monthly to other options, including being able to set it up themselves. One participant shared that

I would say a base for me I would say a monthly reminder would be kind of nice. Anything sooner than that, unless I preference it sooner, would be irritating.

34 years old, white, Miami

Another voiced that the frequency should depend on his current sex life and related behaviors:

It would depend, if I’m in a monogamous relationship, because I don’t need a message. I don’t need them. I’m not going outside that relationship. If I’m playing around or if there’s consensual nonmonogamy, then, yeah, I think it would be beneficial if not every 60 days, maybe every 90 just to be a little extra safe. That’s what I was doing when I was way more active, it’s like every three months.

37 years old, white, Miami

In general, though, many preferred being able to control or customize how and specifically when they receive the messages:

What I would most prefer to be able to do as far as having a scheduled reminder would be just tell it to customize when it would notify me.

19 years old, white, Miami

#### Recommended HIV Testing Intervals With Predetermined Dates

Universally, participants liked this component idea for the testing app. Specifically, some liked to be informed about when to test for HIV:

I really, really like that idea. It would make me even more likely to actually use an app like this. It would give me the tools to know when would be a smart interval to get tested on.

19 years old, white, Miami

Others thought this idea was nice because it made their test decision easy for them:

That’s easy and to me, easy is a good thing.

28 years old, white, Minneapolis

#### Details About HIV Testing Locations and Testing Locator

Participants thought the app must have information beyond where to just get tested for HIV locally:

Just the location is definitely important, how you’re going to get there is definitely important, transportationwise. Information about each testing site is also important, maybe you can add a review sort of thing to each testing site.

23 years old, Asian, Miami

Others also expressed that this component of the testing app should use the GPS that smartphones have:

Maybe the ability to maybe use, like utilize location services and find where is a place to get tested near me.

27 years old, biracial, Minneapolis

#### Monitoring Personal Behaviors

Participants’ opinions varied about including a feature that would allow monitoring of their sexual behaviors in the proposed testing app. Some men liked the idea to help them learn about their own behaviors over time with respect to health and prevention:

For me, it would be fine to have that information there. It’s healthy, it’s educational at the same time, to remind you what you’re doing, or what you’re not. I would use it. Definitely, yes, my friends will [use it].

26 years old, white Hispanic, Miami

Other men indicated they would most likely not use this feature yet understood why it could be appealing to others:

I don’t know if I would honestly use something like that. In the way of putting in my conquests, I guess, into an app. I don’t feel like I would have a use or case for that necessarily. I get the appeal of it and I can see the use or case but in my personal use and case, I don’t see myself using it.

24 years old, white, Minneapolis

In addition, others thought this feature should be optional:

Optional—It’s a lot like the calorie counting programs. You’re only going to do it if you want to and you’re interested in your own progress, but again, that’s something that can put your life into perspective. If you’re actually following it and you see, “Wow, I need to calm down. I’m putting myself at risk,” that’s a good thing, but it should be optional, not something that you’re forced to do but just something that if you want to do it, you can just to keep an eye on yourself.

34 years old, white Hispanic, Miami

## Discussion

### Principal Findings and Recommendations

Findings from this study identified features and functions that MSM identified as influencing them to download and continually use an app over time on their smartphone, as well as some key components of what they would want in an app that aimed to promote regular testing for HIV. Results from this study showed both unique and overlapping themes related to app preferences (ie, general preferences and HIV prevention content) when compared with findings from focus group conducted in prior studies with MSM [15,17-19] Below, we discuss major findings and how they are similar to and unique compared with these existing studies.

Men in our study noted that cost, security, and efficiency were important reasons that influenced whether they downloaded an app onto their smartphone. Perceiving the app to be useful, one of necessity, and positively reviewed by others were additional reasons men described as being important to influencing not only whether they download an app, but also whether to continue to use it over time. Goldenberg and colleagues [17,19] noted that MSM in their studies also prioritized security, the usefulness of the app, and ease of navigation as important factors. Additionally, others have noted that peers are an important influence on app use [15]. Uniquely to this study, we found that men preferred free or very low-cost apps, which may be important for widespread dissemination of HIV testing and prevention apps. Taken together, this and prior studies suggest that developmental work of future HIV prevention app interventions should consider how best to incorporate these critical considerations into the app development process. For example, in addition to usual usability testing, formative work during the alpha and beta testing phases of HIV-related apps may benefit from asking target users to reflect on whether the app helps them to more efficiently address their HIV prevention needs, which features and functions of the app are most useful and necessary to help them reduce their risk for HIV, and whether they perceive the app to be secure for maintaining their private information. The results of our study suggest that future HIV prevention apps must meet these basic requirements for MSM to trust and use an app for any length of time.

Men in our study believed that, in order for them to keep an app and continually use it over longer periods of time, the app must work properly (ie, be reliable), be easy to use, and not be constantly updated. Ease of use has been described as an important consideration for continued app use in at least one prior study [17]. In addition, our results draw attention to the need to ensure that HIV testing and prevention apps work consistently as intended and do not overburden MSM with frequent updates. Relatedly, men in this sample were likely to delete an app from their phone that did not work properly, or they became bored with the app and sought another app that better met their needs. Many of these reasons for using or deleting apps from their phone are intuitive and emphasize the expectations that MSM hold about mobile technologies. For these reasons, and in addition to pilot testing to ensure the functions of an app are working correctly, formative work should ensure that men report the app as being easy to use. Along these lines, researchers may want to consider developing HIV prevention apps that include navigation functions (eg, drop-down menus, home navigation buttons) found in other frequently used apps to help with MSM’s perceptions of ease of use. Such formative research may benefit from using the ISR framework described by Schnall et al [15] or the iterative, community-driven process described by Goldenberg et al [19].

As HIV testing continues to be an important avenue to prevent onward transmission of HIV among MSM, mobile apps aimed at increasing the frequency of HIV testing are needed. To best tailor mobile apps to MSM, we asked men in the focus groups to reflect on their preferences for the following features and functions of an HIV testing smartphone app: HIV test reminders, recommended testing intervals with dates, details about testing locations with an HIV test locator, and monitoring personal sexual behaviors. Overall, men especially appreciated the idea of receiving a reminder to test for HIV, particularly if it was tailored to them and helped reduce the burden of finding locations to test for HIV locally and elsewhere by capitalizing on and integrating with the GPS features found in smartphones. Many of the preferred features and functions of an HIV testing app described by men in this study reflect those in prior studies, especially personalizing the app and providing GPS-enabled HIV testing information [15,17,19]. However, participants’ opinions varied about certain aspects of the proposed HIV testing app, particularly about the self-monitoring of their personal sexual behaviors and how they would receive updates from the app. The same ambivalence about monitoring personal sexual encounters was expressed by men in at least one prior study [19]. These variations in opinions highlight the need to provide options within the HIV testing app that allow users to select delivery option(s) and how often they would like to be notified by the app. Additionally, the self-monitoring of one’s sexual behaviors may aid in increasing HIV testing rates among MSM if this component is viewed as educational, rather than merely a log of their sexual activities. Behavioral self-monitoring aligns with the concept of self-regulation from social cognitive theory [23], which may be used as a theoretical guide to develop future HIV testing apps.

With respect to the development of mobile apps to encourage MSM to test for HIV at regular intervals, researchers should also consider how best to align individuals’ risk behaviors for HIV with the current recommended HIV testing intervals of 3, 6, and 12 months. A variety of options exist for this alignment and will depend on the components of the specific HIV testing app. Moreover, newer options for HIV prevention now exist for MSM, which may positively help affect their frequency of HIV testing. The advent of oral preexposure prophylaxis (PrEP)—a regimen of antiretrovirals taken by those who are HIV-negative to prevent the acquisition of HIV—offers a promising means of prevention. Recent clinical trials have demonstrated the safety of PrEP [24-27] and significant efficacy for HIV prevention. To be most effective, the use of PrEP requires MSM to be tested for HIV every 3 months and to be seen by their medical provider; thus, uptake of PrEP would also help encourage MSM to test for HIV at regular intervals (eg, every 3 months).

### Limitations

This study has important limitations to acknowledge. The results from these focus groups are not intended to be generalizable to all English-speaking, HIV-negative MSM who are smartphone owners in the United States. In addition, participants lived in 2 large urban cities and, therefore, app preferences by other samples of MSM in the Unites States, including those in rural locales and other locations in the South, may differ from this study’s sample. Recall bias may have also affected MSM’s ability to accurately identify features and functions that influenced them to download and use their smartphone app over time. Due to the qualitative, cross-sectional nature of the work conducted, causal inference is not possible; as such, longitudinal research methods should be used in future studies to more accurately illuminate the reasons that motivate MSM to download and use apps on their smartphones. Furthermore, future research should assess smartphone app preferences among a more geographically diverse sample of MSM to determine whether similar attitudes are expressed. These results are meant to be first steps in more fully understanding the needs of HIV-negative MSM with respect to smartphone apps to encourage regular HIV testing.

### Conclusions

This study highlights findings obtained from formative work about the features and functions of smartphone apps that HIV-negative MSM consider when deciding to download mobile apps to their phone and continue to use or not use them over time. Our findings also reflect what components participants would want in an app that promotes regular HIV testing. Themes captured from this study reiterate the importance of formative work to help enhance uptake and sustained use of smartphone apps for HIV prevention, including the promotion of regular testing for HIV. Given the complexity and potential barriers of encouraging HIV-negative MSM to test more regularly for HIV, future smartphone app interventions should also be guided by this and other formative research [15,17-19] and theories of behavior change [28].

## Abbreviations

GPS: global positioning system
HIV: human immunodeficiency virus
ISR: Information Systems Research
MSM: men who have sex with men
PrEP: preexposure prophylaxis
